# Non-alcoholic fatty liver disease in mice with hepatocyte-specific deletion of mitochondrial fission factor

**DOI:** 10.1007/s00125-021-05488-2

**Published:** 2021-05-29

**Authors:** Yukina Takeichi, Takashi Miyazawa, Shohei Sakamoto, Yuki Hanada, Lixiang Wang, Kazuhito Gotoh, Keiichiro Uchida, Shunsuke Katsuhara, Ryuichi Sakamoto, Takaya Ishihara, Keiji Masuda, Naotada Ishihara, Masatoshi Nomura, Yoshihiro Ogawa

**Affiliations:** 1 Department of Medicine and Bioregulatory Science, Graduate School of Medical Sciences, Kyushu University, Fukuoka, Japan; 2 Department of Clinical Chemistry and Laboratory Medicine, Graduate School of Medical Sciences, Kyushu University, Fukuoka, Japan; 3 Department of Protein Biochemistry, Institute of Life Science, Kurume University, Fukuoka, Japan; 4 Department of Biological Science, Graduate School of Science, Osaka University, Osaka, Japan; 5 Section of Oral Medicine for Children, Division of Oral Health, Growth and Development, Faculty of Dental Science, Kyushu University, Fukuoka, Japan; 6 Division of Endocrinology and Metabolism, Department of Internal Medicine, Kurume University School of Medicine, Fukuoka, Japan; 7 Japan Agency for Medical Research and Development, CREST, Tokyo, Japan

**Keywords:** ER stress, Lipid metabolism, MFF, Mitochondrial dynamics, NASH

## Abstract

**Aims/hypothesis:**

Mitochondria are highly dynamic organelles continuously undergoing fission and fusion, referred to as mitochondrial dynamics, to adapt to nutritional demands. Evidence suggests that impaired mitochondrial dynamics leads to metabolic abnormalities such as non-alcoholic steatohepatitis (NASH) phenotypes. However, how mitochondrial dynamics are involved in the development of NASH is poorly understood. This study aimed to elucidate the role of mitochondrial fission factor (MFF) in the development of NASH.

**Methods:**

We created mice with hepatocyte-specific deletion of MFF (*Mff*LiKO). *Mff*LiKO mice fed normal chow diet (NCD) or high-fat diet (HFD) were evaluated for metabolic variables and their livers were examined by histological analysis. To elucidate the mechanism of development of NASH, we examined the expression of genes related to endoplasmic reticulum (ER) stress and lipid metabolism, and the secretion of triacylglycerol (TG) using the liver and primary hepatocytes isolated from *Mff*LiKO and control mice.

**Results:**

*Mff*LiKO mice showed aberrant mitochondrial morphologies with no obvious NASH phenotypes during NCD, while they developed full-blown NASH phenotypes in response to HFD. Expression of genes related to ER stress was markedly upregulated in the liver from *Mff*LiKO mice. In addition, expression of genes related to hepatic TG secretion was downregulated, with reduced hepatic TG secretion in *Mff*LiKO mice in vivo and in primary cultures of MFF-deficient hepatocytes in vitro. Furthermore, thapsigargin-induced ER stress suppressed TG secretion in primary hepatocytes isolated from control mice.

**Conclusions/interpretation:**

We demonstrated that ablation of MFF in liver provoked ER stress and reduced hepatic TG secretion in vivo and in vitro. Moreover, *Mff*LiKO mice were more susceptible to HFD-induced NASH phenotype than control mice, partly because of ER stress-induced apoptosis of hepatocytes and suppression of TG secretion from hepatocytes. This study provides evidence for the role of mitochondrial fission in the development of NASH.

**Graphical abstract:**

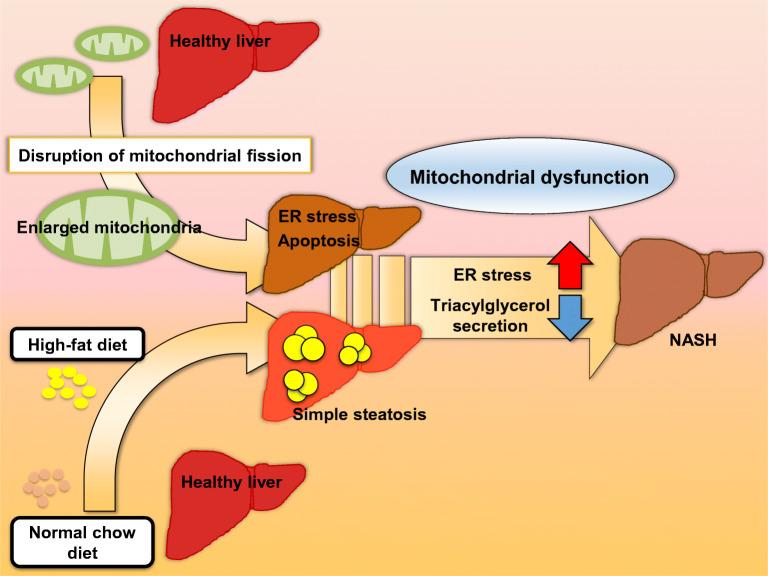

**Supplementary Information:**

The online version contains peer-reviewed but unedited supplementary material available at 10.1007/s00125-021-05488-2.

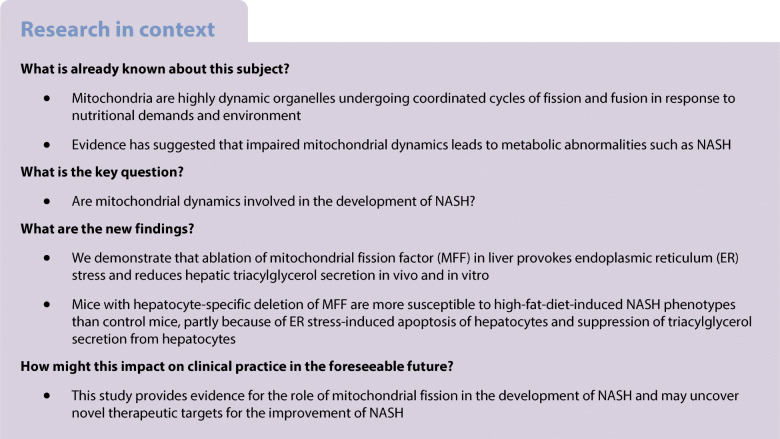



## Introduction

Non-alcoholic fatty liver disease (NAFLD) is characterised by increased accumulation of lipids in the liver without a history of excessive alcohol consumption or known liver disease. The early stage of NAFLD is simple steatosis (without ballooning of hepatocytes, inflammation and fibrosis), known to progress to an aggressive form, non-alcoholic steatohepatitis (NASH), in 10–25% of cases [1–3]. The detailed molecular mechanisms involved in the progression of simple steatosis to NASH are largely unknown.

Mitochondria are unique multifunctional organelles that are critically involved in biological processes such as apoptosis, calcium signalling, ATP production and cellular metabolism [4–7]. They are highly dynamic organelles undergoing coordinated cycles of fission and fusion, known as ‘mitochondrial dynamics’; this is an adaptive response to cellular demands and the environment to maintain metabolic homeostasis [5, 8, 9]. For instance, cellular nutrient excess induces the recruitment of dynamin-related protein-1 (DRP1) to the mitochondrial outer membrane where mitochondrial fission factor (MFF) serves as an adaptor, resulting in membrane constriction and fission via a GTP hydrolysis-related mechanism [5, 10–14]. Mitofusin-2 (MFN2) and optic atrophy 1 (OPA1) are essential proteins for mitochondrial fusion, located at the outer and inner membranes, respectively [15–17]. Mitochondrial functions are also regulated by the structural and functional crosstalk with other organelles, particularly with endoplasmic reticulum (ER). Dynamic contact sites, called mitochondria-associated ER membranes (MAMs), are essential for maintaining mitochondrial fission and fusion, thereby participating in the regulation of ER stress and lipid metabolism [18, 19].

Given that mitochondrial fission-related proteins such as DRP1 and MFF are induced in the liver in high-fat diet (HFD)-induced obese mice [20, 21], it is likely that impaired mitochondrial dynamics are involved in the pathogenesis of NAFLD [4]. On the other hand, there are several reports that enlarged and swollen mitochondria (termed megamitochondria) with loss of their cristae, appear in both humans and mouse models of NASH [22–27]. These observations, taken together, suggest that mitochondrial dynamics play a critical role in the pathogenesis of NAFLD/NASH. However, whether and how mitochondrial dynamics are involved in the progression of simple steatosis to NASH remains poorly understood.

In this study, we created mice with hepatocyte-specific deletion of MFF and performed histological analysis of livers from mice fed normal chow diet (NCD) or HFD. We also examined the changes in the morphology and function of mitochondria in hepatocytes of those mice. Moreover, using primary hepatocytes from those mice, we explored the underlying mechanisms of how impaired mitochondrial fission is involved in the development of NASH.

## Methods

### Animal experiments

**Fig. 1:**
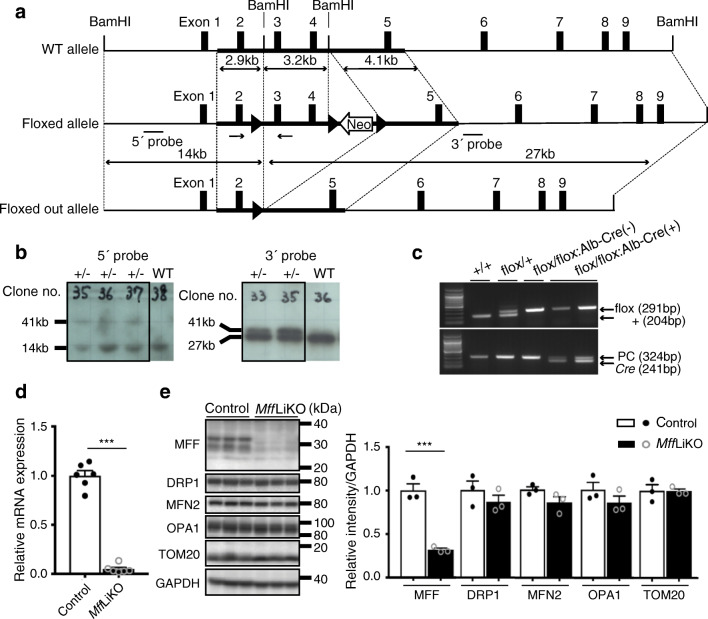
Generation of *Mff*LiKO mice. (**a**) Map of *Mff*-targeting constructs, showing the wild-type (WT) allele (*Mff*^+^), the conditional floxed allele (*Mff*^flox^) and the conditional floxed out allele after *Cre*-mediated recombination (*Mff*^−^). Each exon is represented by a box, and arrowheads indicate the *loxP* sites. The black bars under the line representing the floxed allele indicate the location of the probe used in Southern blot analysis. The black arrows under the line representing the floxed allele indicate the location of a pair of primers used in PCR genotyping. The white arrow indicates a neomycin-resistant cassette (Neo). (**b**) Southern blot analysis of ES cell clones after BamHI digestion and hybridisation with the 5′ or 3′ probe. (**c**) Identification of PCR genotyping with genomic tail DNA. We defined *Mff*^flox/flox^ [shown in the figure as flox/flox:*Alb-Cre*(–)] as control and *Mff*^flox/flox^:*Alb*-*Cre* [shown in the figure as flox/flox:*Alb-Cre*(+)] as *Mff*LiKO. PC indicates positive control. (**d**) mRNA expression of *Mff* in the livers from 30-week-old NCD-fed *Mff*LiKO and control mice (*n*=6 per group) by RT-qPCR. mRNA levels are normalised to *Gapdh*. (**e**) Western blot analysis of protein expression related to mitochondrial dynamics in the livers from 14-week-old NCD-fed *Mff*LiKO and control mice (fed ad libitum). Bar graph shows intensities of each protein band, quantified by densitometric analyses. Each intensity is normalised to GAPDH. Values are expressed as means ± SEM. ***p<0.001, calculated by Student’s *t* test (**d**, **e**). TOM20, translocase of outer membrane

To construct the *Mff*-targeting vector, a DNA fragment (3.2 kb) containing exons 3 and 4 of *Mff* was isolated from a 129S/v mouse genome and inserted into the BamHI site (Fig. 1a). A 2.9 kb fragment flanking exon 2 as the 5′ arm and a 4.1 kb fragment flanking exon 5 as the 3′ arm was inserted into the sites shown in Fig. 1a and subcloned into the pflox vector. The flanked *loxP* sites and a neomycin-resistant cassette are shown in Fig. 1a. Targeted *Mff*^flox/+^ ES clones (no. 35) were injected into C57BL/6J blastocysts to obtain chimeric mice. The *Mff*^flox/+^ mice were backcrossed with C57BL/6J mice for nine generations. They were crossed with *Alb*-*Cre* transgenic mice expressing *Cre* recombinase under the control of the albumin (encoded by *Alb*) promoter B6.Cg-Tg (*Alb*-*Cre*) 21Mgn/J (Stock no. 003574; The Jackson Laboratory, Bar Harbor, ME, USA) to obtain *Mff*LiKO mice. See Electronic supplementary material (ESM) Methods for details of housing and husbandry. For more information on randomisation of the samples, see animal experiments section in ESM Methods.

### Metabolic and biochemical studies

Food intake, locomotor activity and oxygen consumption ($\dot{V}{\mathrm{O}}_2$) of mice were measured individually. Glucose and ITTs were performed as described [28]. Hepatic triacylglycerol (TG) secretion was evaluated with tyloxapol as described [28]. Hepatic TG content was evaluated according to the Folch method [29]. See ESM Methods for further details.

### Histological analysis

The livers were dissected from mice, fixed with 4% (wt/vol.) paraformaldehyde (pH 7.4, 4°C) and embedded in paraffin. Sliced specimens were stained using H&E and Picrosirius Red [28]. We evaluated NASH by NAFLD activity score [30] and fibrosis score [31]. In addition, we used immunostaining of F4/80 and TUNEL assay. See ESM Methods for further details.

### Electron microscopic analysis

The liver samples were perfused with a half Karnovsky fixative and cut into small blocks, post-fixed with 1% (vol./vol.) reduced osmium, dehydrated and embedded in Epon resin as described [28] with some modifications. The mitochondrial size, the width of mitochondrial cristae and the length of MAM [32] were all measured using the free image analysis software (ImageJ 1.52a; NIH, USA). See ESM Methods for further details.

### Microarray analysis

The mRNA from livers of *Mff*LiKO and control mice was subjected to microarray analysis. See ESM Methods for further details.

### Quantitative real-time PCR

Quantitative real-time PCR was used to determine the relative expression levels of mRNAs. Primers used in this study are listed in ESM Table 1. See ESM Methods for further details.

### Western blot analysis

The liver samples were obtained from 14-week-old mice. Western blot analysis was performed as described [28]. The primary and secondary antibodies used are listed in ESM Table 2. See ESM Methods for further details.

### Mouse primary hepatocyte experiments

Mouse primary hepatocytes were isolated by the collagenase perfusion method as described [33] with some modifications. Isolated hepatocytes were cultivated for one day in DMEM supplemented with 11% (vol./vol.) FBS, 1 μmol/l human short-acting insulin, 1 mmol/l l-ascorbic acid and antibiotics. Detailed methods for mitochondrial function assays and TG secretion assays can be found in ESM Methods.

### Isolated mitochondria experiments

The mitochondria from hepatocytes were isolated as described [34]. The respiration rate was measured as oxygen consumption rate [35]. See ESM Methods for further details.

### Statistical analysis

Data are expressed as mean ± SEM, and *p* < 0.05 was considered statistically significant. Statistical analysis was performed using two-tailed Student’s *t* test, two-way ANOVA and repeated measures two-way ANOVA was performed with Bonferroni post hoc test using GraphPad Prism version 6.0 (GraphPad, USA). Histological analyses were blinded and performed by a technical assistant. Otherwise, blinding was not carried out. Data from mice that died in accidents were excluded.

## Results

### Generation of *Mff*LiKO mice

To generate *Mff*LiKO mice, we constructed the floxed allele by inserting the *loxP* sequences into introns flanking exons 3 and 4 of the *Mff* gene (Fig. 1a). Correct initial targeting was confirmed by Southern blotting of genomic DNA from ES cell clones (Fig. 1a, b). We crossed the *Mff* floxed mice and *Alb*-*Cre* transgenic mice. For genotyping, the *Cre* recombinase gene and *loxP*-containing region of the *Mff* gene were amplified by PCR using genomic DNA prepared from tail samples (Fig. 1c). In this study, all *Mff*LiKO mice were born alive and survived similarly to control mice.

The expression of *Mff* was reduced by approximately 10% in the liver from *Mff*LiKO mice relative to control mice (Fig. 1d). There was no appreciable difference between *Mff*LiKO and control mice in other tissues, including brain and heart, where *Mff* is expressed abundantly (ESM Fig. 1a). Western blot analysis revealed a marked reduction of MFF protein expression in the liver isolated from *Mff*LiKO mice (Fig. 1e). The expression of other proteins related to mitochondrial dynamics (DRP1, MFN2, OPA1, and translocase of outer membrane, a protein located in mitochondrial outer membrane) showed no appreciable difference between the genotypes (Fig. 1e, ESM Fig. 1b).

### *Mff*LiKO mice fed NCD exhibit enlarged mitochondria without mitochondrial dysfunction in hepatocytes

**Fig. 2:**
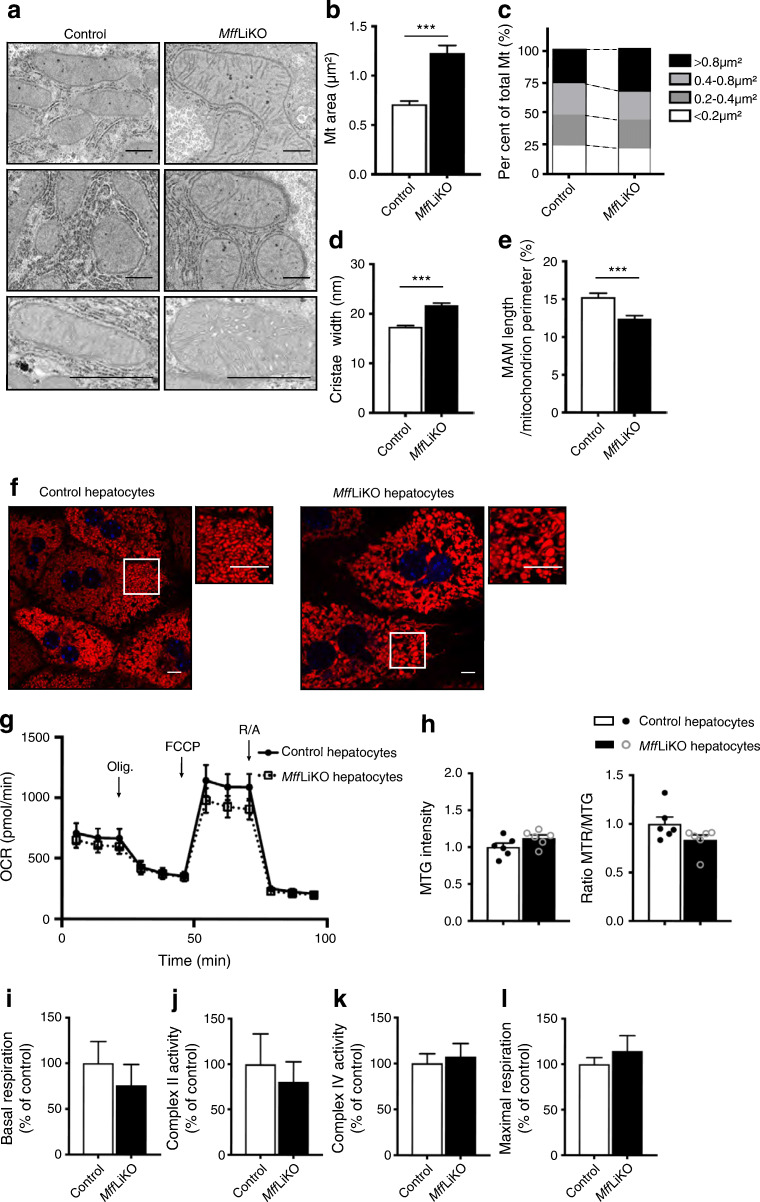
Morphology and function of mitochondria in *Mff*LiKO mice. (**a**) Electron microscopy images from livers of 11-week-old NCD-fed *Mff*LiKO and control mice (*n*=3 per group) (fed ad libitum). Cristae are shown at higher magnification. Scale bar, 1μm. (**b**) Mitochondrial (Mt) areas. (**c**) Distribution of mitochondrial areas is shown as a percentage of mitochondria measured: <0.2 μm^2^ (white bars); 0.2–0.4 μm^2^ (dark grey bars); 0.4–0.8 μm^2^ (light grey bars); or >0.8 μm^2^ (black bars). These cut-off values represent the quartiles. (**d**) Cristae width of mitochondria. (**e**) MAM length per mitochondrion perimeter. (**f**) Immunofluorescence analyses of primary hepatocytes isolated from each phenotype. Representative images from *Mff*LiKO and control mouse hepatocyte imaging of mitochondria (red, MTR) and nuclei (blue, Hoechst33342) are shown, together with high-magnification images of the area denoted by the white box. Scale bar, 10 μm. (**g**) Seahorse XF Cell Mito Stress Test data for oxygen consumption rate (OCR) in hepatocytes isolated from *Mff*LiKO and control mice. Arrows indicate injections into media of the specific stressors oligomycin (Olig.), carbonyl cyanite-4 (trifluoromethoxy) phenylhydrazone (FCCP) and rotenone/antimycin A (R/A) (*n*=5 per group). (**h**) MMP is shown by the ratio of MTR to MTG fluorescence in hepatocytes from *Mff*LiKO and control mice (*n*=6 per group). (**i**–**l**) Mitochondrial respiratory rates of basal respiration (**i**), complex II (**j**), complex IV (**k**) and maximal respiration (**l**) in mitochondria isolated from *Mff*LiKO and control mouse livers. Values are expressed as means ± SEM. ***p<0.001, calculated by Student’s *t* test (**b**, **d**, **e**, **h**–**l**), fraction of total (**c**) or repeated measures two-way ANOVA (**g**). Data analysis was performed in *Mff*LiKO (*n*=582) and control (*n*=645) mitochondria (**b**–**e**). Data are representative of two (**g**) and three (**h**, **i**–**l**) independent experiments

Electron microscopy analysis revealed that mitochondria exhibit a tubular shape and are enlarged in the liver from *Mff*LiKO mice, with an increased proportion of larger-sized mitochondria (Fig. 2a–c). Although the cristae of mitochondria were not lost, the width of mitochondrial cristae in *Mff*LiKO mice was larger than that in control mice (Fig. 2a, d). The proportion of ER in close contact with mitochondria, termed MAM, relative to the total mitochondrial perimeter in *Mff*LiKO mice was smaller than that in control mice (Fig. 2e).

Mitochondria from *Mff*LiKO mouse hepatocytes were swollen and enlarged relative to control hepatocytes (Fig. 2f). In the Seahorse XF Cell Mito Stress Test, there was no significant difference in hepatocyte basal respiration, maximal respiration or ATP production between the genotypes (Fig. 2g and ESM Fig. 1c). There was also no significant difference in mitochondrial membrane potential (MMP) (Fig. 2h). In experiments with the Oxytherm electrode unit, using mitochondria isolated from liver, there were no significant differences in mitochondrial basal and maximal respiratory rates, complex II and complex IV activities between the genotypes (Fig. 2i–l).

### *Mff*LiKO mice fed NCD do not develop NASH phenotypes

**Fig. 3:**
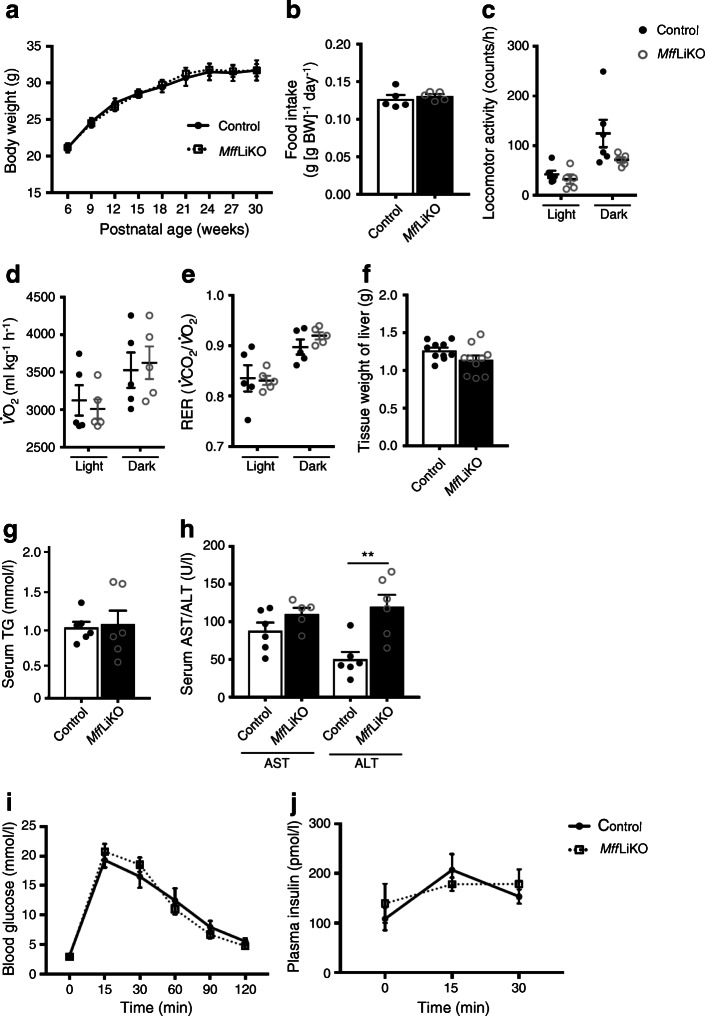
Metabolic phenotypes of 30-week-old NCD-fed *Mff*LiKO mice. (**a**) Growth curve of *Mff*LiKO (*n*=11) and control (*n*=10) mice fed NCD. (**b**) Food intake in *Mff*LiKO and control mice (*n*=5 per group), expressed per g body weight (BW). (**c**) Locomotor activity in *Mff*LiKO (*n*=5) and control (*n*=6) mice. (**d**, **e**) $\dot{V}{\mathrm{O}}_2$ (**d**) and respiratory exchange ratio (RER) (**e**) in *Mff*LiKO and control mice (*n*=5 per group). (**f**) Tissue weight of liver in *Mff*LiKO and control mice (*n*=10 per group). (**g**) Serum TG concentration in fasted *Mff*LiKO and control mice (*n*=6 per group). (**h**) Serum AST and ALT values in *Mff*LiKO (*n*=5 [AST] or 6 [ALT]) and control (*n*=6) mice. (**i**, **j**) Blood glucose (**i**) and plasma insulin (**j**) values during IPGTT of 4- to 6-week-old NCD-fed *Mff*LiKO and control mice (*n*=5 per group). Values are expressed as means ± SEM. **p<0.01, calculated by Student’s *t* test (**a**, **b**, **f**–**h**), two-way ANOVA (**c**–**e**) or repeated measures two-way ANOVA (**i**, **j**)

We found no significant differences in body weight, food intake, locomotor activity, $\dot{V}{\mathrm{O}}_2$ or respiratory exchange ratio between *Mff*LiKO and control mice (Fig. 3a–e). There was also no appreciable difference in liver weight between the genotypes (Fig. 3f). We found a significant increase in serum alanine aminotransferase (ALT) concentrations in *Mff*LiKO mice, although there was no significant difference in serum concentrations of TG and aspartate aminotransferase (AST) between the genotypes (Fig. 3g, h). In the GTT, glucose metabolism and insulin secretion were unaffected in *Mff*LiKO mice (Fig. 3i, j).

**Fig. 4:**
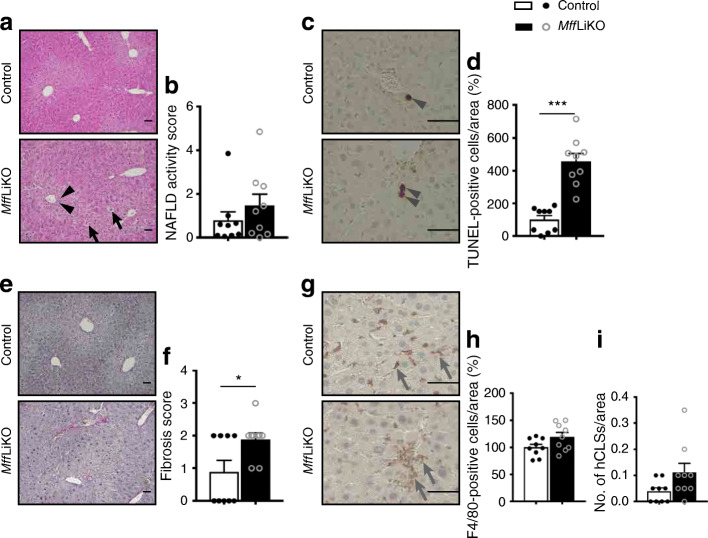
Pathological phenotypes of 30-week-old NCD-fed *Mff*LiKO mice. (**a**) H&E staining in livers from *Mff*LiKO and control mice. Scale bar, 50 μm. (**b**) NAFLD activity score in livers from 30-week-old NCD-fed *Mff*LiKO and control mice (*n*=9 per group). Total score is expressed as follows: 0–2, non-NASH (simple steatosis); 3 or 4, borderline NASH; 5–8, NASH. The score indicates the average of 20 images of liver per mouse in *Mff*LiKO and control mice (*n*=9 per group). (**c**) Apoptotic cells detected by TUNEL assay in livers from *Mff*LiKO and control mice. Scale bar, 50 μm. (**d**) The percentage of TUNEL-positive cells in *Mff*LiKO and control mice (*n*=9 per group). The number of TUNEL-positive cells in *Mff*LiKO mice, calculated as the average of 20 images per liver of each mouse, is presented as the percentage of control mice. (**e**) Picrosirius Red staining in livers from *Mff*LiKO and control mice. Scale bar, 50 μm. (**f**) Fibrosis score in livers from *Mff*LiKO and control mice (*n*=9 per group). (**g**) F4/80 immunostaining in livers from *Mff*LiKO and control mice. Scale bar, 50 μm. (**h**) The percentage of F4/80-positive cells in *Mff*LiKO and control mice (*n*=8 per group). The number of F4/80-positive cells in *Mff*LiKO mice, calculated as the average of 20 images per liver of each mouse, is presented as the percentage of control mice. (**i**) The number of hCLS per area in *Mff*LiKO and control mice (*n*=9 per group). Black arrows indicate ballooning cells, black arrowheads indicate infiltration of inflammatory cells, grey arrowheads indicate TUNEL-positive nuclei and grey arrows indicate F4/80-positive cells. Values are expressed as means ± SEM. *p<0.05 and ***p<0.001, calculated by Student’s *t* test (**b**, **d**, **f**, **h**, **i**)

Histological examination revealed mild infiltration of inflammatory cells and ballooning degeneration of hepatocytes in *Mff*LiKO mice (Fig. 4a, ESM Fig. 1e). There was no increase in lipid accumulation in livers from *Mff*LiKO and control mice (Fig. 4a, ESM Fig. 1e), with no significant difference in hepatic TG content between the genotypes (ESM Fig. 1d). Consequently, we found no significant difference in NAFLD activity score between the genotypes: total score 1.46 vs 0.78, *p =* 0.39; steatosis score 0.69 vs 0.45, *p* = 0.39; lobular inflammation score 0.27 vs 0.039, *p* = 0.091; ballooning score 0.51 vs 0.30, *p* = 0.51 (Fig. 4b and ESM Fig. 1e). The number of TUNEL-positive cells and fibrosis score were significantly increased in *Mff*LiKO mice relative to control mice (Fig. 4c–f). There were no significant differences in the number of F4/80-positive cells and hepatic crown-like structures (hCLSs), where dead or dying hepatocytes are surrounded by macrophages (an origin of hepatic inflammation and fibrosis during the progression from simple steatosis to NASH [36, 37]), between the genotypes (Fig. 4g–i).

Microarray analysis revealed that 769 genes were upregulated in *Mff*LiKO mice. Gene Ontology (GO) analysis revealed that genes related to apoptosis, inflammation and ER stress (e.g. *Mcp-1,* also known as *Ccl2*, encoding monocyte chemoattractant protein-1 and *Atf4* encoding activating transcription factor 4 [ATF4]) were upregulated in *Mff*LiKO mice (ESM Fig. 2a). We confirmed no significant difference in mRNA expression of inflammatory genes (*F4/80* [also known as *Adgre1*], *Tnfa* and *Il6*) between the genotypes (ESM Fig. 2c). Among the ER stress-related genes, *Chop* (also known as *Ddit3*, encoding C/EBP homologous protein [CHOP]), *P8* (also known as *Nupr1*, encoding nuclear protein 1) and *Trib3* (encoding tribbles homologue 3) expression levels did not differ significantly between the groups (ESM Fig. 2d). However, mRNA expression of the gene encoding activating transcription factor 3 (*Atf3*), which is downstream of the protein kinase R-like ER kinase (PERK)–eukaryotic translation factor 2α (eIF2α) pathway, was significantly increased in *Mff*LiKO mice compared with control mice (ESM Fig. 2d). In contrast, the expression levels of *Xbp1* (encoding X-box binding protein 1 [XBP1]) and *Atf6* (encoding activating transcription factor 6 [ATF6]), included in other ER stress pathways, were not increased in *Mff*LiKO mice (ESM Fig. 2d).

### *Mff*LiKO mice fed HFD develop NASH phenotypes

**Fig. 5:**
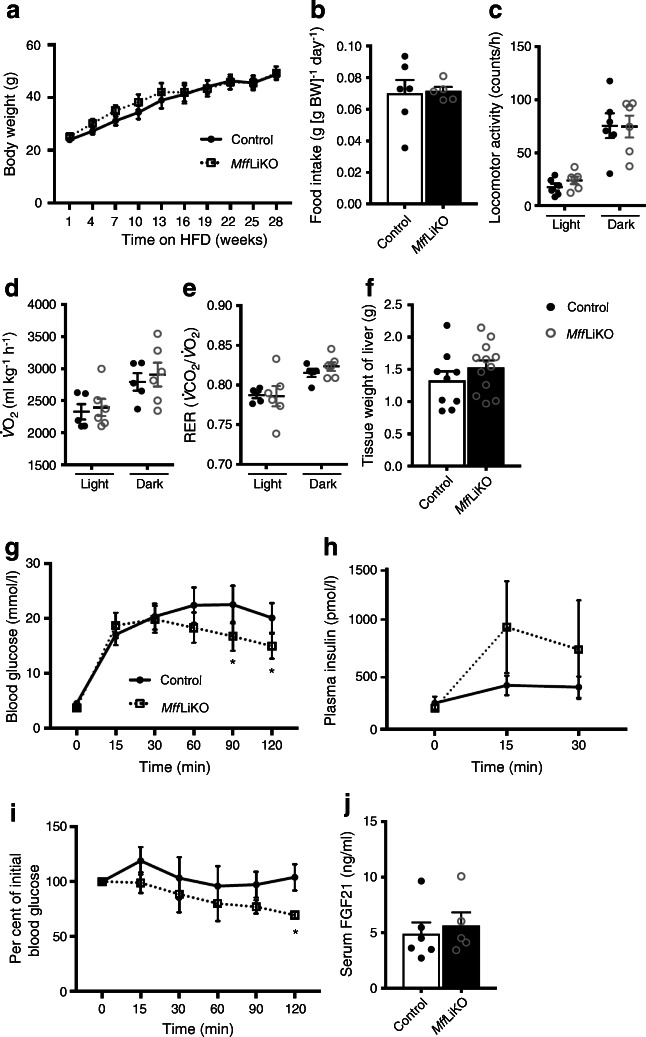
Metabolic phenotypes of 30-week-old HFD-fed *Mff*LiKO mice. (**a**) Growth curve of *Mff*LiKO and control mice (*n*=6 per group) fed HFD from 6–8 weeks of age. (**b**) Food intake in *Mff*LiKO (*n*=5) and control (*n*=6) mice, expressed per g body weight (BW). (**c**) Locomotor activity in *Mff*LiKO and control mice (*n*=6 per group). (**d**, **e**) $\dot{V}{\mathrm{O}}_2$ (**d**) and respiratory exchange ratio (RER) (**e**) in *Mff*LiKO (*n*=6) and control (*n*=5) mice. (**f**) Tissue weight of liver in *Mff*LiKO (*n*=11–12) and control (*n*=8–9) mice. (**g**, **h**) Blood glucose (**g**) and plasma insulin (**h**) values during IPGTT of 30-week-old HFD-fed *Mff*LiKO and control mice (*n*=7 per group). (**i**) Per cent of the initial blood glucose values during ITT of 30-week-old fed *Mff*LiKO (*n*=6) and control (*n*=5) mice. (**j**) Serum FGF21 concentrations of 30-week-old HFD-fed *Mff*LiKO (*n*=5) and control (*n*=6) mice fasted for 17 h. Values are expressed as means ± SEM. *p<0.05, calculated by Student’s *t* test (**a**, **b**, **f**, **j**), two-way ANOVA (**c**–**e**) or repeated measures two-way ANOVA (**g**–**i**)

We next examined the metabolic phenotypes of *Mff*LiKO mice during the HFD feeding. There was no significant difference in body weight, food intake, metabolic variables or liver weight between *Mff*LiKO and control mice (Fig. 5a–f). During IPGTT, blood glucose at 90 and 120 min was decreased in *Mff*LiKO mice compared with control but there was no difference in plasma insulin levels between the genotypes (Fig. 5g, h). Insulin sensitivity was improved slightly in *Mff*LiKO mice (Fig. 5i). The serum concentration of fibroblast growth factor 21 (FGF21), which is reported to be upregulated in mice with hepatocyte-specific deletion of *Drp1* [28], was unchanged in *Mff*LiKO vs control mice (Fig. 5j).

**Fig. 6:**
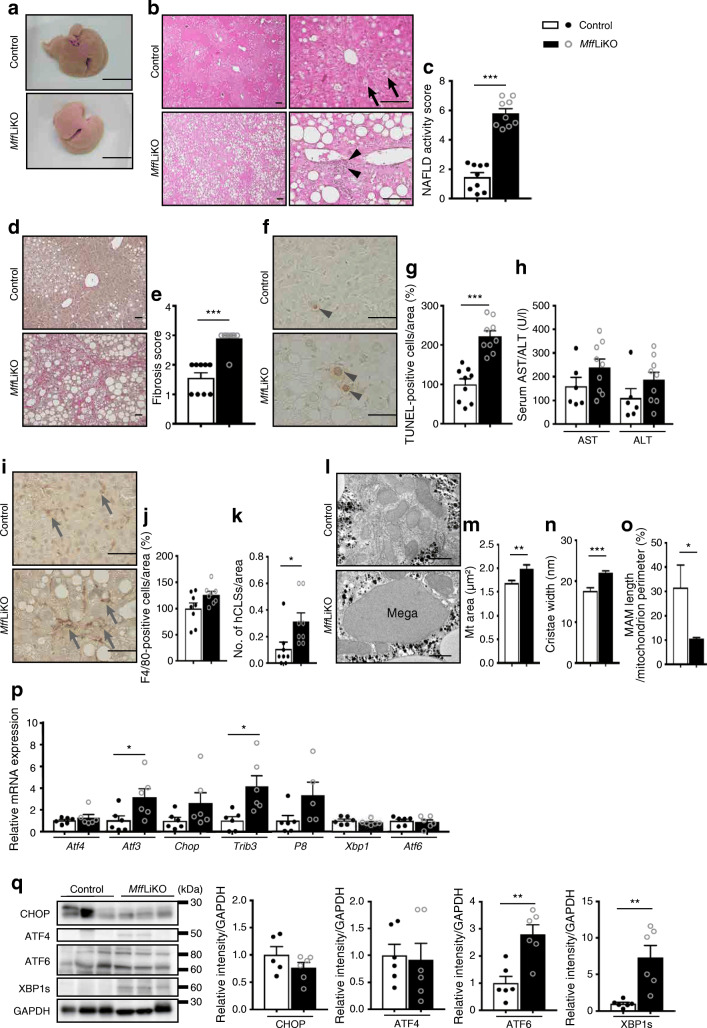
Steatohepatitis is caused in 30-week-old HFD-fed *Mff*LiKO mice. (**a**, **b**) Representative macroscopic images (**a**) and H&E staining (**b**) of livers from *Mff*LiKO and control mice. Scale bar, 1 cm (**a**) or 100 μm (**b**). (**c**) NAFLD activity score. Total score is expressed as follows: 0–2, non-NASH (simple steatosis); 3 or 4, borderline NASH; 5–8, NASH. The score indicates the average of 20 images of liver per mouse in *Mff*LiKO and control mice (*n*=9 per group). (**d**, **e**) Representative images of Picrosirius Red staining (**d**) and fibrosis score (**e**) in livers from *Mff*LiKO and control mice (*n*=9 per group). Scale bar, 50 μm. (**f**, **g**) Representative images of TUNEL assay (**f**) and the percentage of TUNEL-positive cells (**g**) in *Mff*LiKO and control mice (*n*=9 per group). The number of TUNEL-positive cells in *Mff*LiKO mice, calculated as the average of 20 images per liver of each mouse, is presented as the percentage of control mice. Scale bar, 50 μm. (**h**) Serum AST and ALT values in *Mff*LiKO (*n*=9) and control (*n*=6) mice. (**i**, **j**) Representative images of F4/80 immunostaining (**i**) and the percentage of F4/80-positive cells (**j**) in *Mff*LiKO and control mice (*n*=8 per group). The number of F4/80-positive cells in *Mff*LiKO mice, calculated as the average of 20 images per liver of each mouse, is presented as the percentage of control mice. Scale bar, 50 μm. (**k**) The number of hCLSs per area in *Mff*LiKO and control mice (*n*=9 per group). (**l**) Electron microscopy images of livers from 30-week-old HFD-fed *Mff*LiKO and control mice (*n*=3 per group) (refed for 4 h). Scale bar, 1 μm. (**m**) Mitochondrial areas (*Mff*LiKO *n*=687, control *n*=778 mitochondria). (**n**) Cristae width of mitochondria. (**o**) MAM length per mitochondrion perimeter. (**p**) Hepatic mRNA expression related to ER stress in fasted *Mff*LiKO and control mice (for each group *n*=5 or 6). mRNA levels were normalised to *Gapdh* and presented relative to control set at 1 by quantitative PCR. (**q**) Hepatic protein expression related to ER stress in fasted *Mff*LiKO and control mice, detected by western blot analysis (*n*=3 per group). Bar graphs show intensities of each protein band, quantified by densitometric analyses. Each intensity is normalised to GAPDH. Black arrows indicate ballooning cells, black arrowheads indicate infiltration of inflammatory cells, grey arrowheads indicate TUNEL-positive nuclei and grey arrows indicate F4/80-positive cells. ‘Mega’ indicates megamitochondria. Values are expressed as means ± SEM. *p<0.05, **p<0.01 and ***p<0.001, calculated by Student’s *t* test (**c**, **e**, **g**, **h**, **j**, **k**, **m**–**q**). Data analysis was performed in *Mff*LiKO (*n*=687) and control (*n*=778) mitochondria (**m–o**). Data are representative of three independent experiments (**q**)

Upon macroscopic inspection, there was a clear increase in lipid accumulation in liver from *Mff*LiKO mice relative to control mice (Fig. 6a). Histological analysis revealed macrovesicular steatosis with ballooning degeneration of hepatocytes and massive infiltration of inflammatory cells in the liver from *Mff*LiKO mice (Fig. 6b). NAFLD activity score was markedly increased in *Mff*LiKO mice relative to control mice: total score 5.81 vs 1.47, *p* < 0.001; steatosis score 1.39 vs 0.52, *p* < 0.001; lobular inflammation score 2.43 vs 0.16, *p* < 0.001; ballooning score 1.98 vs 0.79, *p* < 0.001 (Fig. 6b, c and ESM Fig. 1e). Fibrosis score was also significantly increased in *Mff*LiKO mice compared with control mice (score 2.89 vs 1.56, *p* < 0.001) (Fig. 6d, e). The number of TUNEL-positive apoptotic cells in *Mff*LiKO mice was higher than that in control mice (Fig. 6f, g). Serum AST and ALT concentrations did not differ significantly between *Mff*LiKO and control mice (Fig. 6h). The number of F4/80-positive macrophages was not significantly increased in *Mff*LiKO mice relative to control mice (Fig. 6i, j), although the number of hCLSs was significantly increased (*p* < 0.05) (Fig. 6k).

Microarray analysis revealed that 882 genes were upregulated in *Mff*LiKO mice relative to control mice during HFD feeding. GO analysis revealed that genes related to inflammation and ER stress, such as *Mcp-1* and *Chop*, were upregulated in *Mff*LiKO mice (ESM Fig. 3a). We found increased expression of mRNAs for inflammatory genes (*F4/80*, *Mcp-1*, and *Tnfa*) and fibrogenic genes (*Col1a1*, encoding collagen type 1 α1, and *Timp1*, encoding tissue inhibitor of metalloproteinase 1) in *Mff*LiKO mice (ESM Fig. 3c, d). Expression of ER stress-related genes included in the PERK–eIF2α pathway, such as *Atf3* and *Trib3* was increased, while expression of *Xbp1* and *Atf6* was not increased in *Mff*LiKO mice relative to control mice (Fig. 6p). Western blot analysis revealed that there were no significant differences in protein expression of CHOP and ATF4 between the genotypes. However, protein expression of ATF6 and spliced XBP1 (XBP1s) was significantly increased in *Mff*LiKO mice relative to control mice (Fig. 6q). In microarray analysis, 627 genes were downregulated in *Mff*LiKO mice relative to control mice, with genes related to lipid metabolism (e.g. *Srebp1c* [also known as *Srebf1*], encoding sterol regulatory element-binding transcription factor 1c, and *Acsl1*, encoding Acyl-CoA synthetase long-chain family member 1) being highlighted in GO analysis (ESM Fig. 3b).

### Enlarged mitochondria in *Mff*LiKO mice fed HFD exhibit impaired mitochondrial function

In electron microscopic analysis, the mean mitochondrial size was increased in *Mff*LiKO mice, where megamitochondria, the extremely large and aberrantly shaped mitochondria frequently observed in NASH [22–24], were diffusely distributed (Fig. 6l, m). The width of mitochondrial cristae in *Mff*LiKO mice was also larger than that in control mice (Fig. 6n). The proportion of MAM length relative to total mitochondrial perimeter was significantly smaller in the livers of *Mff*LiKO mice than in control mice (Fig. 6o).

To assess mitochondrial function, we examined the mitochondrial respiration, MMP, and mitophagy using primary hepatocytes from HFD-fed *Mff*LiKO and control mice. In the Seahorse XF Cell Mito Stress Test, the oxygen consumption rate was decreased in *Mff*LiKO mouse hepatocytes relative to control mouse hepatocytes (ESM Fig. 4a). Next, we evaluated MMP using MMP-insensitive dye (MTG) and MMP-sensitive dye (MTR). The fluorescence intensity of MTG was significantly increased in *Mff*LiKO mouse hepatocytes relative to control mouse hepatocytes (ESM Fig. 4b). In addition, the fluorescence intensity of MTR was also significantly decreased in *Mff*LiKO mouse hepatocytes (ESM Fig. 4b). It is thus conceivable that damaged mitochondria accumulate in *Mff*LiKO mouse hepatocytes. Finally, we examined the accumulation of mitophagy intermediates by double-immunostaining with antibodies against p62 and pyruvate dehydrogenase. We found that the fluorescence intensity of p62 was increased in *Mff*LiKO mouse hepatocytes relative to control mouse hepatocytes (ESM Fig. 4c). Furthermore, p62 was mostly co-localised in mitochondria in *Mff*LiKO mouse hepatocytes. These findings suggest that mitophagy intermediates accumulated in liver from *Mff*LiKO mice through the combination of MFF deletion and HFD loading.

### Deletion of *Mff* suppresses hepatic TG secretion in vivo and in vitro

**Fig. 7:**
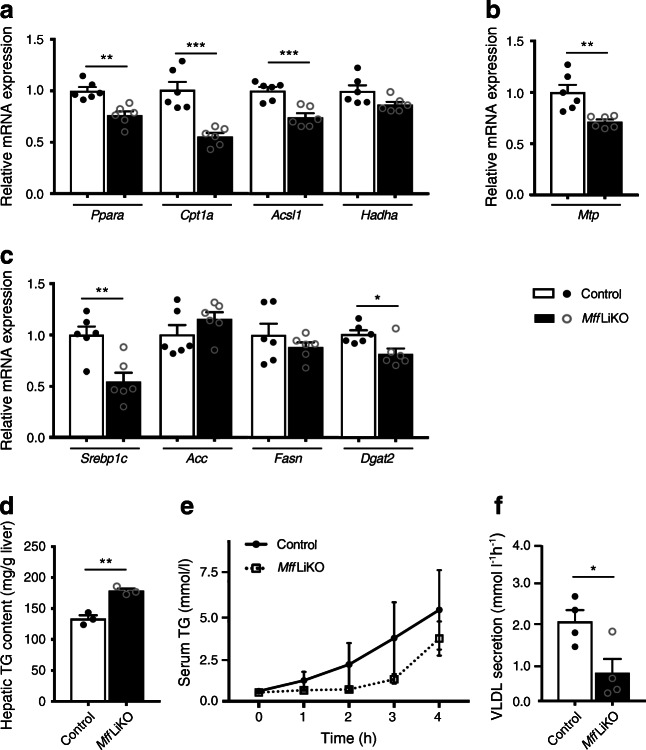
Lipid metabolism profile in 30-week-old HFD-fed *Mff*LiKO mice. (**a**–**c**) Hepatic mRNA expression in fasted *Mff*LiKO and control mice refed for 4 h (*n*=6 per group). mRNA levels were normalised to *Gapdh* and presented relative to control set at 1 by quantitative PCR. Genes related to fatty acid oxidation (**a**), TG secretion from liver (**b**) and lipogenesis (**c**) are shown. (**d**) Hepatic TG content in *Mff*LiKO and control mice refed for 4 h after 17 h fasting (*n*=3 per group). (**e**, **f**) Evaluation of hepatic TG secretion (**e**) and VLDL secretion per 1 h (**f**) after fasting for 4 h with tyloxapol in 30-week-old HFD-fed *Mff*LiKO and control mice (*n*=4 per group). Values are expressed as means ± SEM. *p<0.05, **p<0.01 and ***p<0.001, calculated by Student’s *t* test (**a**–**d**, **f**) or repeated measures two-way ANOVA (**e**). *Dgat2* encodes diacylglycerol O-acyltransferase 2 and *Hadha* encodes hydroxyacyl-CoA dehydrogenase trifunctional multienzyme complex subunit-α

Next, we examined lipid metabolism in the liver from *Mff*LiKO mice. Fatty acid β-oxidation genes, such as *Ppara* (encoding peroxisome proliferator-activated receptor-α), *Cpt1a* (encoding carnitine palmitoyltransferase 1a) and *Acsl1*, were significantly downregulated in *Mff*LiKO mice relative to control mice (Fig. 7a). Expression of *Mtp* (also known as *Mttp*, encoding microsomal TG transfer protein [MTP]), which is important for the assembly and secretion of VLDL from the liver [38], was significantly decreased in *Mff*LiKO mice relative to control mice (Fig. 7b), as was the expression of *Srebp1c*, a key transcription factor of de novo lipogenesis (Fig. 7c). However, there was no significant difference in expression of other lipogenic genes such as *Acc* (encoding acetyl coenzyme A carboxylase 1) and *Fasn* (encoding fatty acid synthase) (Fig. 7c). These observations suggest that fatty acid oxidation and TG secretion is reduced in the liver of *Mff*LiKO mice.

**Fig. 8:**
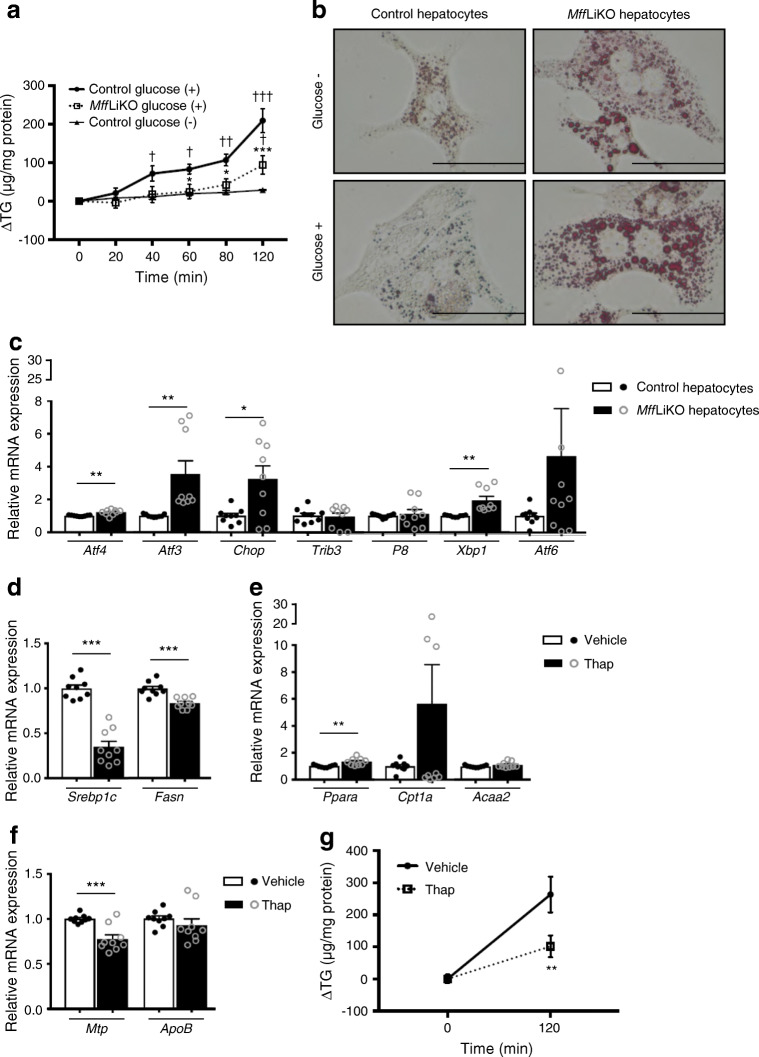
Association of TG secretion and ER stress in primary hepatocytes. (**a**, **b**) Profile of TG secretion assay in *Mff*LiKO and control mouse hepatocytes. The time course of TG content of supernatant fraction (**a**), showing data for control mouse hepatocytes stimulated with 25 mmol/l glucose (*n*=5), *Mff*LiKO mouse hepatocytes stimulated with 25 mmol/l glucose (*n*=3) and control mouse hepatocytes without glucose (*n*=3). Oil Red O staining images before and 120 min after the later glucose stimulation in *Mff*LiKO and control mouse hepatocytes; scale bar, 50 μm (**b**). (**c**) mRNA expression of genes related to ER stress in *Mff*LiKO and control mouse hepatocytes (*n*=9 per group). mRNA levels were normalised to *Gapdh* and presented relative to control set at 1 by quantitative PCR. (**d**–**f**) mRNA expression of genes related to lipogenesis (**d**), fatty acid oxidation (**e**) and TG secretion from liver (**f**) in hepatocytes treated with vehicle or 450 mmol/l thapsigargin (Thap) for 12 h (*n*=9). mRNA levels were normalised to *Gapdh* and presented relative to control set at 1 by quantitative PCR. (**g**) Profile of TG secretion assay in primary cultured hepatocytes treated with vehicle or 450 mmol/l Thap for 12 h. The time course of TG contents of supernatant fraction after stimulation with 25 mmol/l glucose. Data are representative of three independent experiments. Values are expressed as means ± SEM. *p<0.05, **p<0.01 and ***p<0.001 for indicated comparisons, calculated by Student’s *t* test (**c**–**f**), or for *Mff*LiKO-glucose (+) group vs control-glucose (+) group (**a**) or Thap group vs vehicle group (**g**), calculated by repeated measures two-way ANOVA (**a**, **g**). ^†^*p*<0.05, ^††^*p*<0.01 and ^††††^*p*<0.001 for control-glucose (+) group compared with control-glucose (−) group. *Acc2* encodes acetyl-coenzyme A acyltransferase 2

To clarify why postprandial TG content was significantly increased in the liver of *Mff*LiKO mice relative to control mice (Fig. 7d), we measured the TG accumulation rate in serum after i.p. injection of tyloxapol, a lipoprotein lipase inhibitor. Because serum TG concentrations are determined by TG secretion from the liver and lipoprotein lipase-mediated TG uptake in peripheral tissues under fasting conditions, serum TG concentrations after treatment with tyloxapol represented in vivo hepatic TG secretion [39]. Hepatic TG secretion was suppressed by about 60% in *Mff*LiKO mice fed HFD for 30 weeks (Fig. 7e, f). Furthermore, we performed a TG secretion assay using primary hepatocytes isolated from *Mff*LiKO and control mice. We examined glucose-stimulated TG secretion from primary hepatocytes and confirmed that TG secretion is induced in response to glucose stimulation of control mouse hepatocytes (Fig. 8a), consistent with the findings of a previous report [40]. Glucose-stimulated TG secretion was markedly reduced in *Mff*LiKO mouse hepatocytes (Fig. 8a). The lipid droplets remained in *Mff*LiKO mouse hepatocytes even 120 min after glucose stimulation (Fig. 8b).

The expression of genes related to ER stress, such as *Atf4*, *Atf3* and *Chop*, was increased in *Mff*LiKO mouse hepatocytes relative to control hepatocytes (Fig. 8c). To explore the causal relationship between increased ER stress and suppressed TG secretion, we treated control mouse hepatocytes with thapsigargin. Thapsigargin treatment downregulated the genes related to de novo lipogenesis (*Srebp1c*, *Fasn*) and upregulated the genes related to fatty acid β-oxidation (*Ppara*) significantly (Fig. 8d, e). Importantly, expression of genes related to TG secretion, such as *Mtp*, was significantly decreased in response to thapsigargin, although there was no change in *ApoB* expression (Fig. 8f). Glucose-stimulated TG secretion was markedly reduced in thapsigargin-treated control mouse hepatocytes relative to secretion following vehicle treatment (Fig. 8g).

## Discussion

Evidence has accumulated suggesting that mitochondrial dynamics play a role in the pathogenesis of NASH [27]. However, it remains to be determined whether or, if any, how mitochondrial fission is associated with the disease progression from simple steatosis to NASH. Because mice with global deletion of *Mff* die early in life (unpublished data, Nomura M. et al), it is impossible to address the role of mitochondrial dynamics in the pathogenesis of NASH. In this study, we created mice with hepatocyte-specific deletion of *Mff* and found that they are apparently healthy, with aberrant mitochondrial morphologies only in hepatocytes. Although abnormal mitochondrial morphologies are reported in fibroblasts derived from individuals harbouring mutations in *MFF* [41], it remains unclear whether MFF is involved in the pathogenesis of NASH. We found no appreciable difference in the abundance of other proteins related to mitochondrial dynamics between the genotypes, suggesting that the phenotypes observed are due solely to *Mff* deficiency. Therefore, *Mff*LiKO mice provide a unique experimental system with which to assess the role of MFF in the pathogenesis of NASH.

It is a matter of debate whether altered mitochondrial morphology is the cause of mitochondrial dysfunction and metabolic abnormality. It was reported that enlargement of mitochondria per se causes the insufficiency of mitophagy (the selective autophagy of mitochondria), leading to lipid accumulation in liver [27]. On the other hand, a report described that the restoration of mitochondrial fragmentation by genetically manipulating the expression of OPA1 improves neither mitochondrial dysfunction nor lipid accumulation in liver [42]. In this study, we demonstrate that hepatocyte-specific deletion of *Mff* causes abnormal mitochondrial morphologies, thereby leading to apoptosis of hepatocytes, inflammation and fibrosis, although *Mff*LiKO mice fed NCD do not develop NASH phenotypes. However, *Mff*LiKO mice show severe ER stress and develop NASH phenotypes during HFD feeding, while control mice do not. Recently, the ‘multiple parallel hit’ hypothesis, whereby multiple factors, such as cytokines, oxidative stress, mitochondrial dysregulation, lipotoxicity and ER stress, contribute concurrently to the progression of NASH, has become widely accepted [43]. According to this hypothesis, the development of NASH requires both environmental and genetic factors. It was reported that the dysfunction of mitochondrial dynamics induces apoptosis, inflammation and fibrosis of hepatocytes [14, 44]. These findings suggest that morphological abnormalities of mitochondria per se might be involved in the progression of NASH as one of the multiple factors.

There is considerable evidence that hepatic mitochondria are functionally and morphologically altered in response to nutritional demands. Evidence has suggested that mitochondrial fission is stimulated and thereafter round-shaped small-sized mitochondria appear in mouse liver during HFD feeding [20]. It was also reported that the expression of mitochondrial fission-related proteins, such as DRP1 and MFF, is increased in mouse liver during HFD feeding [20]. Hammerschmidt et al recently reported that *Mff* deficiency attenuates fatty acid-induced mitochondrial fragmentation in vitro and in vivo, although whether MFF is involved in the development of NAFLD was not addressed [45]. Moreover, several reports have described that mitochondria are swollen and enlarged, with reduced cristae or megamitochondria, in livers from NASH model mice and individuals with NASH [23, 28]. When mice were fed HFD for a long period, the expression of *Mff* in liver was decreased (ESM Fig. 4d). Mitochondria play a role in alleviating lipotoxic responses due to fat accumulation but continuous fat accumulation may cause them to lose the capacity to overcome increased NEFA concentration in the advanced stage of the disease. It is therefore conceivable that impaired mitochondrial fission to adapt to excess energy was followed by severe aberrant mitochondrial morphologies and vulnerability to the hepatic compensation mechanism against liver damage with severe hepatic steatosis.

This study is the first demonstration that *Mff* deficiency results in impaired hepatic TG secretion both in vivo and in vitro. We found that gene expression of *Atf3* and *Trib3* and protein expression of ATF6 and XBP1s are upregulated in HFD-fed *Mff*LiKO mice, suggesting that ER stress is induced during HFD feeding. The discrepancy between mRNA and protein expression of some genes may be due to the post-transcriptional regulation of those genes. It was reported that mitochondrial dysfunction may cause ER stress, with activation of the unfolded protein response (UPR), leading to activation of the de novo lipogenic pathway and the aggravation of steatosis [46]. Prolonged ER stress or chronic activation of the UPR also induces hepatocyte death and inflammation via the CHOP-dependent pathway [47]. ER stress is well known to be involved in TG accumulation in hepatocytes [48]. Moreover, tunicamycin, an antibiotic that induces ER stress, decreases TG secretion through activation of the eIF2α pathway [49]. In this study, we found that the expression of *Mtp* was downregulated in *Mff*LiKO mice and that TG secretion from *Mff*LiKO mouse hepatocytes was decreased. Furthermore, we showed that genes related to ER stress are upregulated in *Mff*LiKO mouse hepatocytes and that glucose-stimulated TG secretion was reduced in thapsigargin-treated hepatocytes, suggesting that increased ER stress could decrease TG secretion from *Mff*LiKO mouse hepatocytes. These observations suggest that *Mff* deficiency results in abnormal mitochondrial morphologies, thereby inducing ER stress, which may contribute to impaired hepatic TG secretion through the downregulation of MTP.

In this study, we demonstrated that glucose metabolism is unchanged in *Mff*LiKO mice, which is in sharp contrast with our previous report that the disruption of mitochondrial fission in the liver protected mice from diet-induced obesity and that metabolic deterioration improved glucose metabolism in mice with targeted disruption of *Drp1* in the liver [28]. The serum concentration of FGF21, which is known to increase energy expenditure and fat utilisation, was increased in mice with liver-specific deletion of *Drp1* while it was unchanged in *Mff*LiKO mice. Given that MFF is one of the receptors of DRP1, DRP1 might induce more severe ER stress than MFF, and glucose metabolism might be controlled by other receptors of DRP1. Further studies are required to elucidate how mitochondrial fission is involved in the regulation of glucose metabolism.

In conclusion, this study provides evidence that impaired mitochondrial fission induces ER stress and reduces hepatic TG secretion, which may play a key role in the pathogenesis of NASH. Our data suggest that impaired mitochondrial fission increases the risk of developing NASH during the period of excess energy, potentially leading to the development to the full-blown NASH phenotypes.

## Supplementary information

ESM (PDF 757 kb)

## Data Availability

The data are available from the corresponding author on reasonable request.

## Abbreviations

ALT: Alanine aminotransferase
AST: Aspartate aminotransferase
ATF4: Activating transcription factor 4
ATF6: Activating transcription factor 6
CHOP: C/EBP homologous protein
DRP1: Dynamin-related protein-1
eIF2α: Eukaryotic translation factor 2α
ER: Endoplasmic reticulum
FGF21: Fibroblast growth factor 21
hCLS: Hepatic crown-like structures
HFD: High-fat diet
MAM: Mitochondria-associated ER membrane
MFF: Mitochondrial fission factor
MFN2: Mitofusin-2
MMP: Mitochondrial membrane potential
MTG: MitoTracker Green
MTR: MitoTracker Red
MTP: Microsomal TG transfer protein
NAFLD: Non-alcoholic fatty liver disease
NASH: Non-alcoholic steatohepatitis
NCD: Normal chow diet
OPA1: Optic atrophy 1
PERK: Protein kinase R-like ER kinase
TG: Triacylglycerol
UPR: Unfolded protein response
XBP1: X-box binding protein 1
XBP1s: Spliced XBP1
