# Supplementary material for: Non-alcoholic fatty liver disease in mice with hepatocyte-specific deletion of mitochondrial fission factor
Source: Diabetologia. 2021 May 29;64(9):2092–107. doi: 10.1007/s00125-021-05488-2 (PMC8382662; doi:10.1007/s00125-021-05488-2)
Supplement: Supplementary file 1 — (PDF 757 kb) [file 125_2021_5488_MOESM1_ESM.pdf]

Electronic Supplementary Material

ESM Methods

Animal experiments

Male C57BL/6J wildtype (WT) mice were purchased from KBT Oriental Co., Ltd. (Saga, Japan). The animals were housed in a temperature-, humidity-, and light-controlled room (12-h light and 12-h dark cycle) and allowed free access to water and normal chow diet (NCD) (CRF-1; Oriental Yeast, Tokyo, Japan), unless otherwise noted. For high-fat diet (HFD) feeding experiments, 6-8-week-old male mice were fed HFD (D12451; 385 kcal/100 g, 45% energy as fat; Research Diets, Inc., New Brunswick, NJ) for 30 weeks. At the end of the experiments, the animals were sacrificed under anesthesia. All animal experiments were performed in accordance with the Guide for the Care and Use of Laboratory Animals and approved by the Ethics Committees on Animal Experimentation of Kyushu University Graduate School of Medicine (A19-088-0). The animals were randomly divided into NCD and HFD feeding groups and kept for 30 weeks.

Metabolic and biochemical studies

Food intake was measured by Multi Feeder (#MF-1; SHINFACTORY, Fukuoka, Japan). Locomotor activity was measured using ACTIMO (SHINFACTORY). $\dot{V}O_2$ and respiratory exchange ratio (RER) were measured using Oxymax (Columbus Instruments, Columbus, OH). Glucose and insulin tolerance tests were performed as described [1]. Serum triacylglycerol (TG), aspartate aminotransferase (AST), and alanine aminotransferase (ALT) concentrations were measured using the respective standard enzymatic assays. Hepatic TG secretion was evaluated after fasting for 4 h with a lipoprotein lipase (LPL) inhibitor tyloxapol as described [1]. Total lipids in the liver were extracted and measured according to the Folch method [2]. Serum fibroblast growth factor

21 (FGF21) concentrations were measured using enzyme-linked immunosorbent assay kit (R&D Systems, Minneapolis, MN, USA).

Histological analysis

The paraffin-embedded liver specimens were sliced to five-μm-thick sections and stained using hematoxylin-eosin (H&E) and Picrosirius red [1]. H&E staining was used for the determination of NAFLD activity score, which is defined as the sum of hepatic steatosis (0-3), lobular inflammation (0-3), and hepatocyte ballooning (0-2); ranging from 0 to 8 as described [3]. Fibrosis score was quantified according to the method of Brunt et al. [4]. For immunostaining, rat anti-mouse F4/80 (1:100 dilution; Bio-Rad Laboratories, Inc., Hercules, CA) was used. The number of hepatic crown-like structures (hCLS), dead or dying hepatocytes surrounded by macrophages considered as an origin of hepatic inflammation and fibrosis, was counted as described [5]. For TUNEL assay, liver sections were stained with ApopTag (R) Plus Peroxidase In Situ Apoptosis Detection Kit (Merck KGaA, Darmstadt, Germany) according to the manufacturer's instruction. Sections were analyzed using a BZ-8000 microscope and BZ- II Analyzer exe ver. 1.31 (Keyence, Osaka, Japan).

Electron microscopic analysis

The liver was dissected from mice fed NCD for 11 weeks and those fed HFD for 30 weeks after fasting overnight plus refeeding for 4 h. The samples were perfused with a half Karnovsky fixative; 2% (vol./vol.) paraformaldehyde (PFA) and 2.5% (vol./vol.) glutaraldehyde. The tissues were cut into small blocks, post-fixed with 1% (vol./vol.) reduced osmium and embedded in Epon resin as described [1] with some modifications. The samples were observed with a transmission electron microscope (H-7650; Hitachi

High-Technologies Co., Ibaraki, Japan). Data analysis was performed with 35-52 sections for mice fed NCD (*Mff*LiKO and control mice; n = 3 each) and 36 sections for those fed HFD (*Mff*LiKO and control mice; n = 3 each). The mitochondrial size, the width of mitochondrial cristae, and the length of mitochondria-associated ER membranes (MAM) with the distance between endoplasmic reticulum (ER) and mitochondria being within 30 nm [6] were all measured using the free image analysis software (ImageJ 1.52a, NIH, USA).

Microarray analysis

Total RNA was extracted from the liver and purified using SV Total RNA Isolation System (Promega Corporation, Madison, WI). Microarray analysis was performed by SurePrint G3 Mouse Gene Expression Microarray 8×60K v2 (Agilent Technologies, Santa Clara, CA). The result was analysed using Feature Extraction Software version 9.5.1.1 (Agilent Technologies, Inc.). To identify up-regulated or down-regulated genes, we calculated the Z-scores and ratios (non-log-scaled fold-change) from the normalised signal intensities of each probe and compared to the controls. In this study, Z-score ≥ 2.0 and ratio ≥1.5-fold and Z-score ≤ -2.0 and ratio ≤ 0.66 were considered as up-regulated and down-regulated genes, respectively. To determine significantly over-represented gene ontology (GO) biological process (BP) terms and significant enrichment of pathways, we used Database for Annotation, Visualization and Integrated Discovery (DAVID) version 6.8 (http://david.abcc.ncifcrf.gov/home.jsp).

Quantitative real-time PCR

Total RNA was extracted from the liver tissues and primary hepatocytes of mice. Quantitative real-time PCR analysis was performed as described [1]. The mRNA levels

were normalised to that of *Gapdh* mRNA. Primers used in this study are listed in ESM Table 1.

Western blot analysis

The liver samples were obtained from 14 weeks old mice. Western blot analysis was performed as described [1]. The primary and secondary antibodies used are listed in ESM Table 2.

Mouse primary hepatocyte experiments

Mitochondrial function assays:

For assessment of mitochondrial bioenergetic function, primary hepatocytes isolated from NCD mice (2×10^4 cells/well in 100 μL) or HFD mice (4×10^4 cells/well in 100 μL) were immediately cultured in XFe24 cell microplates overnight. We used the Mito-Stress Test Kit (Agilent Technologies, Inc.) and measured the Oxygen Consumption Rate (OCR) on a XFe24 Extracellular Flux Analyzer (Agilent Technologies, Inc.). Experiments were performed according to the manufacturer's protocol.

For fluorescent signal quantification by a plate reader, primary hepatocytes (1×10^4 cells/well) were seeded onto 96-well plate. They were stained with DAPI, mitochondrial membrane potential (MMP)-insensitive dye, MitoTracker Green, and MMP-sensitive dye, MitoTracker Red. After washing twice with 1×HBSS, fluorescent intensity was measured by a plate reader (Ensight, Perkin Elmer). MMP was evaluated by the ratio of red to green fluorescence intensity.

For live imaging, MitoTracker Red (Thermo Fisher Scientific Inc., Wilmington, DE) and Hoechst 33342 (Dojindo Laboratories, Kumamoto, Japan) were used to stain

mitochondria and nuclei, respectively, and analysed on Zeiss LSM700 laser scanning confocal microscope (Carl Zeiss, Oberkochen, Germany).

For intracellular protein staining with antibodies, primary hepatocytes were fixed with 4% (wt/vol.) paraformaldehyde (Wako Pure Chemical Industries) and permeabilized with 0.2% (vol./vol.) Triton X-100/PBS for 15 min at room temperature. They were incubated at room temperature with anti-p62 (PM045; MBL) and anti-PDH (ab110333; Abcam) antibodies. Image analysis was performed with a TCS SP8 laser-scanning confocal microscope (Leica 331 Microsystems) with a 63x/NA 1.40 oil objective lens.

TG secretion assays:

For TG secretion assay, primary hepatocytes (25×10^4 cells/well) were plated on collagen-coated 6-well plates with growth medium (25 mmol/l glucose, oleic acid-free), washed with the pre-incubation solution (glucose-free Ringer's solution added 300 μmol/l oleic acid) 3 times, and incubated with the pre-incubation solution for 20 minutes. The pre-incubation solution was replaced with Ringer's solution added 300 μmol/l oleic acid with or without 25 mmol/l glucose, after which the supernatant was collected at 0, 20, 40, 60, 80, and 120 minutes. The TG content was measured using TG E-Test Wako (FUJIFILM Wako Pure Chemical Corporation, Osaka, Japan).

Thapsigargin is a non-competitive inhibitor of sarco/endoplasmic reticulum Ca^{2+} ATPase (SERCA), which has often been used as a potent inducer of ER stress. For thapsigargin experiment, primary hepatocytes from control mice (35×10^4 cells/well) were plated on collagen-coated 12-well plates, incubated with serum-depleted DMEM for 3 h, and treated with growth medium (25 mmol/L glucose, oleic acid-free) plus DMSO (Sigma-Aldrich Co.) with or without thapsigargin (450 nmol/L, Merck KGaA) for 12 h. The TG secretion was examined as described above.

For Oil Red O staining, primary hepatocytes (25 × 10^4 cells/well) before and 120 min after glucose stimulation were fixed in 10% (wt/vol.) paraformaldehyde for 10 minutes and treated with 60% (vol./vol.) isopropanol for 1 minute at room temperature. The hepatocytes were incubated with 60% (vol./vol.) Oil red O (#25633-92, Nacalai Tesque) solution in distilled water for 20 minutes.

Isolated mitochondria experiments

The mitochondria from hepatocytes were isolated as described [7]. To measure the respiration rate as oxygen consumption, we used Oxytherm electrode unit (Hansatech Instruments Ltd., Norfolk, UK) as described [8]. In this study, oxygen consumption of isolated mitochondria was measured by administrations of 5 mmol/L glutamate and 5 mmol/L malate for complex I, 10 mmol/L succinate, and 100 μmol/L rotenone for complex II, and 10 mmol/L ascorbate, 400 μmol/L N,N,N’,N’-tetramethyl-p-phenylenediamine, and 20 μmol/L antimycin A for complex IV.

References

1. Wang L, Ishihara T, Ibayashi Y, et al. (2015) Disruption of mitochondrial fission in the liver protects mice from diet-induced obesity and metabolic deterioration. Diabetologia 58(10): 2371-2380. 10.1007/s00125-015-3704-7

2. Zhang Z, Li B, Meng X, et al. (2016) Berberine prevents progression from hepatic steatosis to steatohepatitis and fibrosis by reducing endoplasmic reticulum stress. Sci Rep 6: 20848. 10.1038/srep20848

3. Kleiner DE, Brunt EM, Van Natta M, et al. (2005) Design and validation of a histological scoring system for nonalcoholic fatty liver disease. Hepatology 41(6): 1313-1321. 10.1002/hep.20701

4. Brunt EM, Janney CG, Di Bisceglie AM, Neuschwander-Tetri BA, Bacon BR (1999) Nonalcoholic steatohepatitis: a proposal for grading and staging the histological lesions. Am J Gastroenterol 94(9): 2467-2474. 10.1111/j.1572-0241.1999.01377.x

5. Itoh M, Kato H, Suganami T, et al. (2013) Hepatic crown-like structure: a unique histological feature in non-alcoholic steatohepatitis in mice and humans. PLoS One 8(12): e82163. 10.1371/journal.pone.0082163
6. Giacomello M, Pellegrini L (2016) The coming of age of the mitochondria-ER contact: a matter of thickness. Cell Death Differ 23(9): 1417-1427. 10.1038/cdd.2016.52
7. Wieckowski MR, Giorgi C, Lebiedzinska M, Duszynski J, Pinton P (2009) Isolation of mitochondria-associated membranes and mitochondria from animal tissues and cells. Nat Protoc 4(11): 1582-1590. 10.1038/nprot.2009.151
8. Ishihara T, Ban-Ishihara R, Maeda M, et al. (2015) Dynamics of mitochondrial DNA nucleoids regulated by mitochondrial fission is essential for maintenance of homogeneously active mitochondria during neonatal heart development. Mol Cell Biol 35(1): 211-223. 10.1128/MCB.01054-14

ESM Tables

ESM Table 1 - The primer sequences list of the selected genes for genotyping PCR and quantitative Real-time PCR

For genotyping PCR

**Gene**	**Forward primer（5'-3'）**	**Reverse primer（5'-3'）**
Cre	GCATTTCTGGGGATTGCTTA	CCCGGCAAAACAGGTAGTTA
Mff	GGCAGTGAGACATCACTAGG	GTACAATTGCACTGAGTGAGC
PC	CTAGGCCACAGAATTGAAAGATCT	GTAGGTGGAAATTCTAGCATCATCC

For quantitative Real-time PCR

**Gene**	**Forward primer（5'-3'）**	**Reverse primer（5'-3'）**
Acaa2	GTCCGGATGCGATCCTACTA	GCCTCCACTCACATTGGTTT
Acc	CCAGGCCATGTTGAGACGCT	ATCACAGAGCGGACGCCATC
Acsl1	CAGAACATGTGGGTGTCCAG	GTTACCAACATGGGCTGCTT
ApoB	GGCCTTGATGAAGAAAACCA	ATGCCCCTCTTGATGTTCAG
Atf3	ACCTCCTGGGTCACTGGTATTTG	TTCTTTCTCGCCGCCTCCTTTTCC
Atf4	ACTATCTGGAGGTGGCCAAG	CATCCAACGTGGTCAAGAGC
Atf6	CAGTTGCTCCATCTCCTCTCC	TGGGACACTGGCATTGGTTTG
Chop	AGCCTGGTATGAGGATCTGC	CTCCTGCTCCTTCTCCTTCA
Col1a1	GAGCGGAGAGTACTGGATCG	GTTCGGGCTGATGTACCAGT
Cpt1a	CCAGGCTACAGTGGGACATT	GAACTTGCCCATGTCCTTGT
Dgat2	TACTTCACCTGGCTGGCATT	GTGGTCAGCAGGTTGTGTGT
Fasn	CTCCGTGGACCTTATCACTA	CTGGGAGAGGTTGTAGTCAG
Fis1	GCCTGGTTCGAAGCAAATAC	AACAGCCCTCGCACATACTT
F4/80	TGACAACCAGACGGCTTGTG	GCAGGCGAGGAAAAGATAGTGT
Gapdh	TGCACCACCAACTGCTTAGC	GATGCAGGGATGATGTTC

Hadha	TGACGCTGGTTATCTTGCTG	TGCTGAACGTTCTTCTGTGG
Il-1b	TGTGAAATGCCACCTTTTGA	GGTCAAAGGTTTGGAAGCAG
Il-6	GATGGATGCTACCAAACTGGA	CTCTGAAGGACTCTGGCTTTG
Mcp-1	CCCAATGAGTAGGCTGGAGA	TCTGGACCCATTCCTTCTTG
MiD49	GTCCTACTCGCCCTGTCCTT	CCTAGCACCAATCGAGCATT
MiD51	TTGCCACACTGGCAGTTAAG	CAGCTCCTCTTCCCTGAATG
Mff	GGAGTTCCAAATGCCAGTGT	TGGTGTTTTCAGTGCCAGAG
Mtp	TGAGCGGCTATACAAGCTCAC	CTGGAAGATGCTCTTCTCGC
Ppara	AGTTCGGGAACAAGACGTTG	GGACTTTCCAGGTCATCTGC
P8	ACCAAGAGAGAAGCTGCTGC	CTCCCTCTCCAGAACCTCACT
Srebp1c	GATCAAAGAGGAGCCAGTGC	TAGATGGTGGCTGCTGAGTG
Timp1	GTGGGAAATGCCGCAGAT	GGGCATATCCACAGAGGCTTT
Tnfa	CCACCACGCTCTTCTGTCTA	AGGGTCTGGGCCATAGAACT
Trib3	CCCACAGGCACAGAGTACAC	CGTCCTCTCACAGTTGCTGA
Xbp1	GAACCAGGAGTTAAGAACACG	AGGCAACAGTGTCAGAGTCC

Abbreviations

Acaa2 Acetyl-Coenzyme A acyltransferase 2

Dgat2 Diacylglycerol O-acyltransferase 2

Fis1 Fission, mitochondrial 1

Hadha Hydroxyacyl-CoA dehydrogenase trifunctional multienzyme complex subunit -alpha

MiD49 Mitochondrial dynamic protein of 49 kDa (also known as Mief2)

MiD51 Mitochondrial dynamic protein of 51 kDa (also known as Mief1)

ESM Table 2 - Antibody information for Western blot analysis in this study

**Target Protein (Clonality)**	**Host**	**Provider**	**Catalogue no. (RRID)**	**Dilution**
**Primary antibodies**				
Anti-GAPDH, Monoclonal Antibody, Peroxidase Conjugated (monoclonal antibody)	mouse	FUJIFILM Wako Pure Chemical Corporation (Osaka, Japan)	015-25473 (AB_2665526)	1:5000
ATF-4 (monoclonal antibody)	rabbit	Cell Signaling Technology (Danvers, MA, USA)	11815 (AB_2616025)	1:1000
ATF6 (polyclonal antibody)	rabbit	Merck Millipore (Darmstadt, Germany)	09-069 (AB_1586891)	1:1000
CHOP (monoclonal antibody)	mouse	Cell Signaling Technology (Danvers, MA, USA)	2895 (AB_2089254)	1:1000
DLP1 (also known as DNM1L) (monoclonal antibody)	mouse	BD transduction Laboratories (San Jose, CA, USA)	611112 (AB_398423)	1:2500
FIS1 (polyclonal antibody)	rabbit	Sigma-Aldrich (St. Louis, MO, USA)	HPA017430 (AB_1848608)	1:2500
MFF (polyclonal antibody)	rabbit	Proteintech (Rosemont, IL, USA)	17090-1-AP (AB_2142463)	1:500
MFN2 (monoclonal antibody)	mouse	Abnova (Taipei, Taiwan)	H00009927-M03 (AB_530127)	1:2500

OPA1 (monoclonal antibody)	mouse	BD transduction Laboratories (San Jose, CA, USA)	612606 (AB_399888)	1:5000
Tom20 (monoclonal antibody)	rabbit	Cell Signaling Technology (Danvers, MA, USA)	42406 (AB_2687663)	1:2500
XBP-1s (monoclonal antibody)	rabbit	Cell Signaling Technology (Danvers, MA, USA)	12782 (AB_2687943)	1:1000
**Secondary antibodies**				
Anti-mouse IgG, HRP-linked Antibody (polyclonal antibody)	horse	Cell Signaling Technology (Danvers, MA, USA)	7076 (AB_330924)	1:5000
Anti-rabbit IgG, HRP-linked Antibody (polyclonal antibody)	goat	Cell Signaling Technology (Danvers, MA, USA)	7074 (AB_2099233)	1:5000

Primary antibodies other than GAPDH diluted to Can Get Signal® Solution 1 (TOYOBO CO., LTD., Osaka, Japan). Secondary antibodies diluted to Can Get Signal® Solution 2 (TOYOBO CO., LTD., Osaka, Japan). Dilutions of each antibody without the primary antibody were used as negative controls for validation.

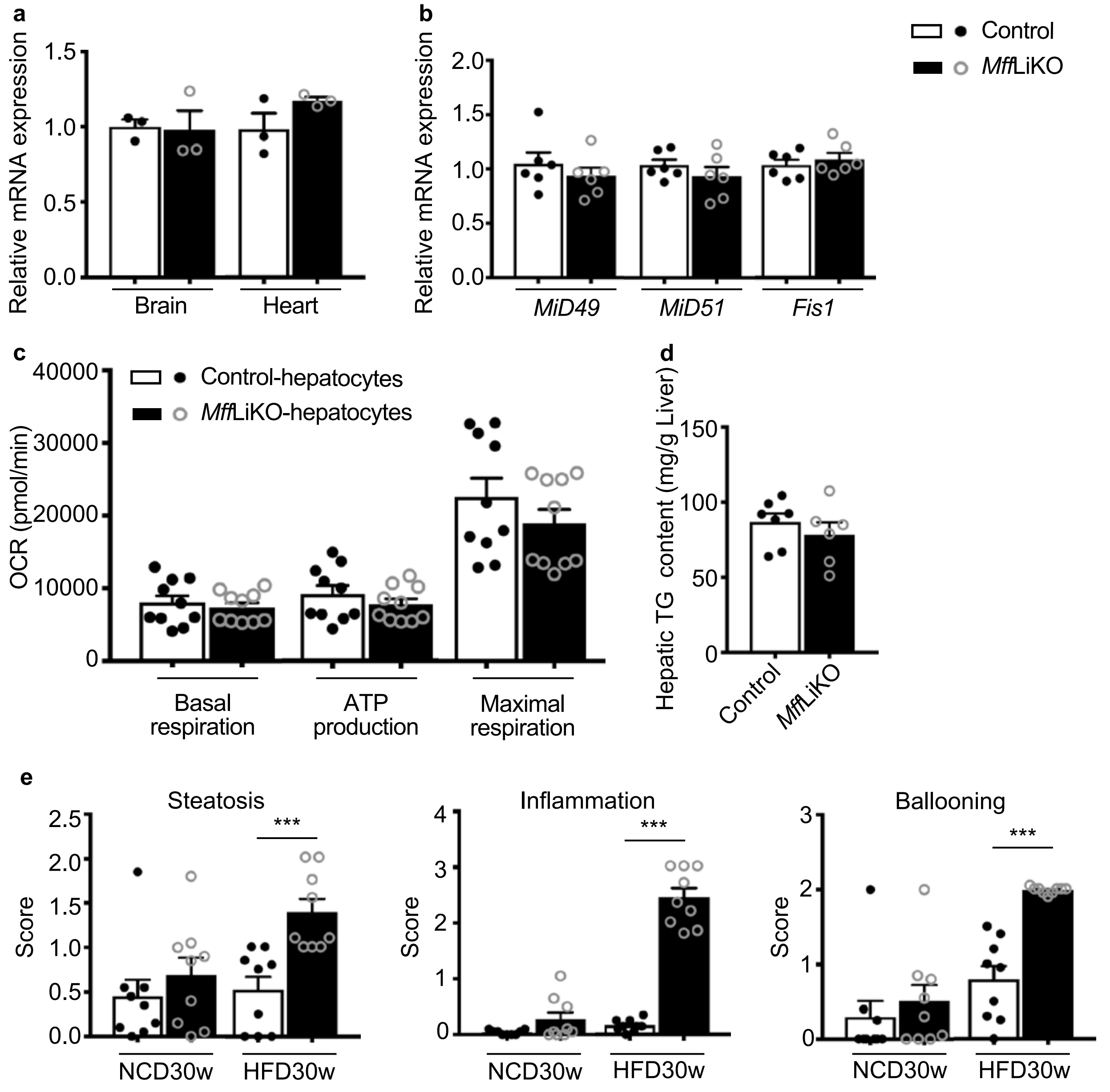

ESM Fig. 1 - Generation of *Mff*LiKO mice and hepatic phenotype in *Mff*LiKO mice.

(**a**) mRNA expression of *Mff* across mitochondria rich brain and heart from 30-week-old NCD-fed *Mff*LiKO and control mice (for each group n = 3) by RT-qPCR. mRNA levels are normalised to *Gapdh*.

(**b**) mRNA expression of mitochondrial fission factors other than *Mff*, such as *MiD49*, *MiD51* and *Fis1*, in the livers from 30-week-old NCD-fed *Mff*LiKO and control mice (for each group n = 6).

(**c**) Mitochondrial basal respiration, ATP production and maximal respiration by Seahorse XF Cell Mito Stress Test in *Mff*LiKO and control mouse hepatocytes. Data are representative of two independent experiments.

(**d**) Hepatic TG contents in the liver from 30-week-old NCD-fed *Mff*LiKO and control mice after 17-hour-fasting (for each group n = 6-7).

(**e**) The breakdown of NAFLD activity score by steatosis, inflammation and ballooning in the liver from 30-week -old NCD-fed or HFD-fed *Mff*LiKO and control mice. The score indicates the average of 20 images of liver per mouse group n = 9.

Values are expressed as means ± SEM. *** p <0.001, calculated by Student' s t test (**a**-**e**). *Fis1* encoding fission, mitochondrial 1, *MiD49* encoding mitochondrial dynamic protein of 49 kDa (also known as Mief2) and *MiD51* encoding mitochondrial dynamic protein of 51 kDa (also known as Mief1).

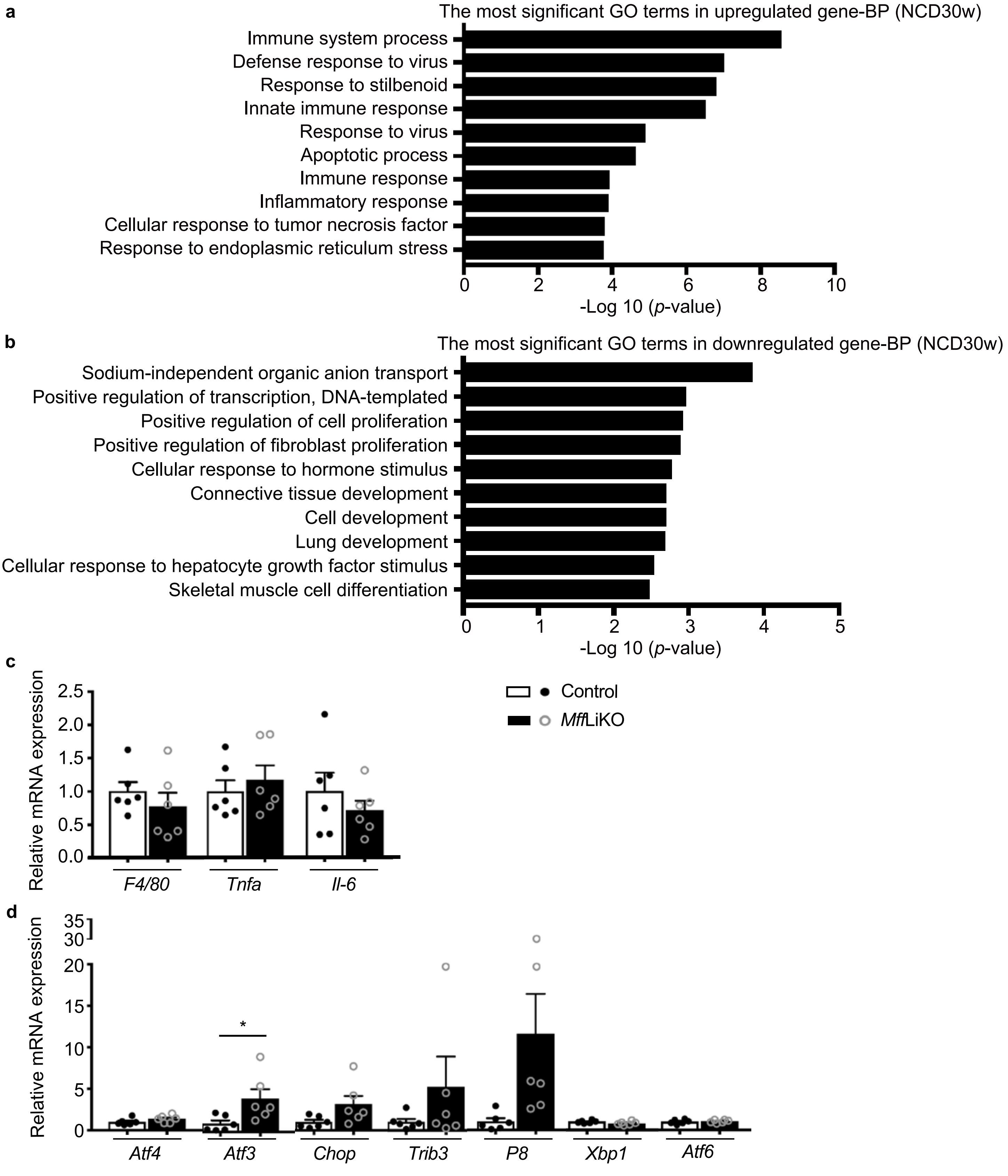

ESM Fig. 2 - The expression of genes in 30-week-old NCD-fed *Mff*LiKO mice.

(**a**, **b**) Microarray analysis in 30-week-old NCD-fed *Mff*LiKO and control mouse liver (for each group *n* =1) (fasted). GO terms in upregulated (**a**) and downregulated (**b**) gene-biological process (BP).

(**c**, **d**) Hepatic mRNA expression in 30-week-old NCD-fed *Mff*LiKO and control fasted mice (for each group *n* =6). mRNA levels were normalised to *Gapdh* and presented relative to control set at 1 by quantitative PCR. The genes related to inflammatory markers (**c**) and ER stress (**d**).

Values are expressed as means ± SEM. * p <0.05, calculated by Student' s t test (**c**, **d**).

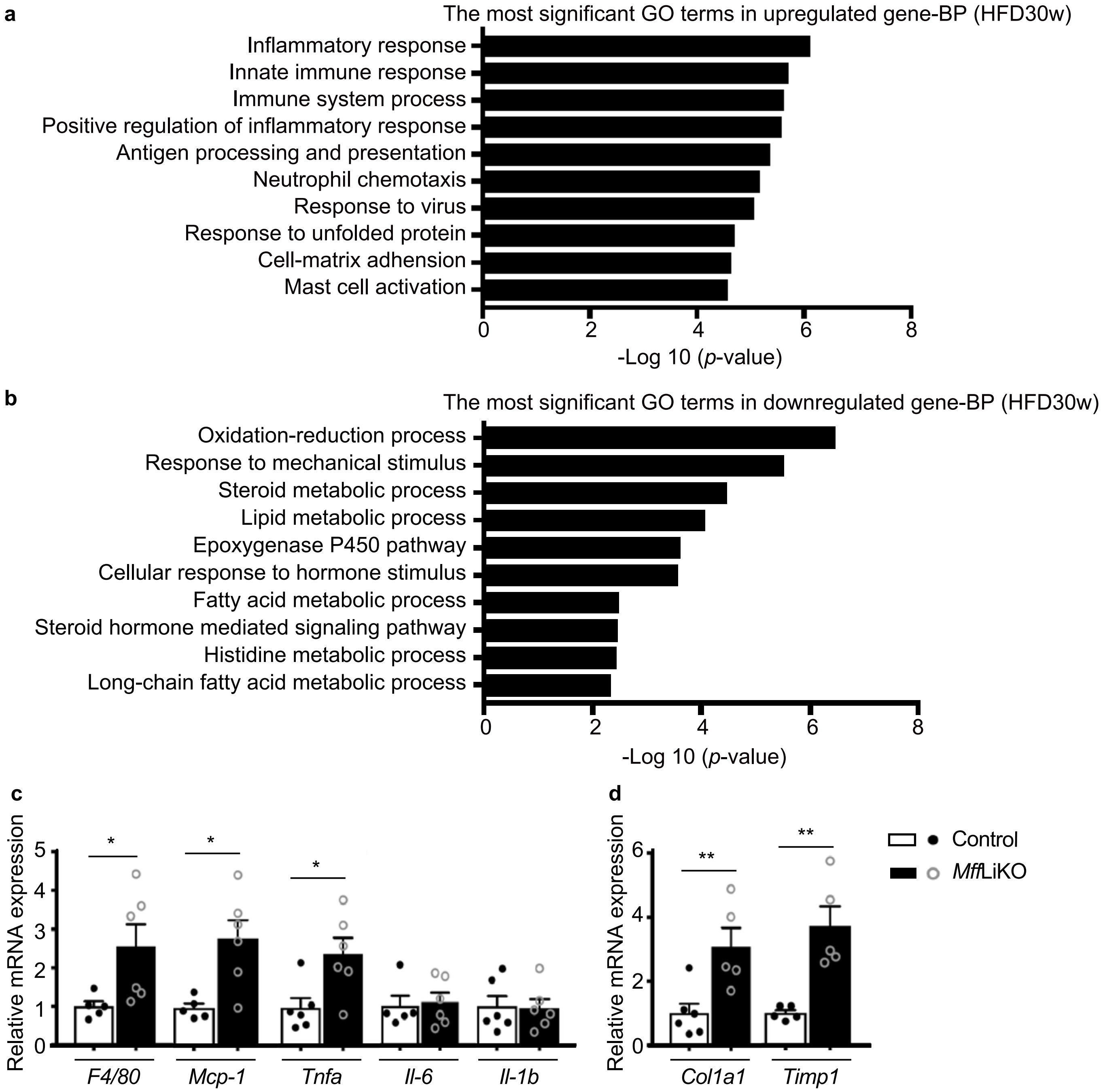

ESM Fig. 3 - The expression of genes in 30-week-old HFD-fed *Mff*LiKO mice.

(**a**, **b**) Microarray analysis in 30-week-old HFD-fed *Mff*LiKO and control mouse liver (for each group n = 3) (fasted). GO terms in upregulated (**a**) and downregulated (**b**) gene-BP.

(**c**, **d**) Hepatic mRNA expression in 30-week-old HFD-fed *Mff*LiKO and control fasted mice (for each group n = 5-6). mRNA levels were normalised to *Gapdh* and presented relative to control set at 1 by quantitative PCR. The genes related to inflammatory markers (**c**) and fibrosis markers (**d**).

Values are expressed as means ± SEM. * p <0.05 and ** p <0.01, calculated by Student' s t test (**c**, **d**).

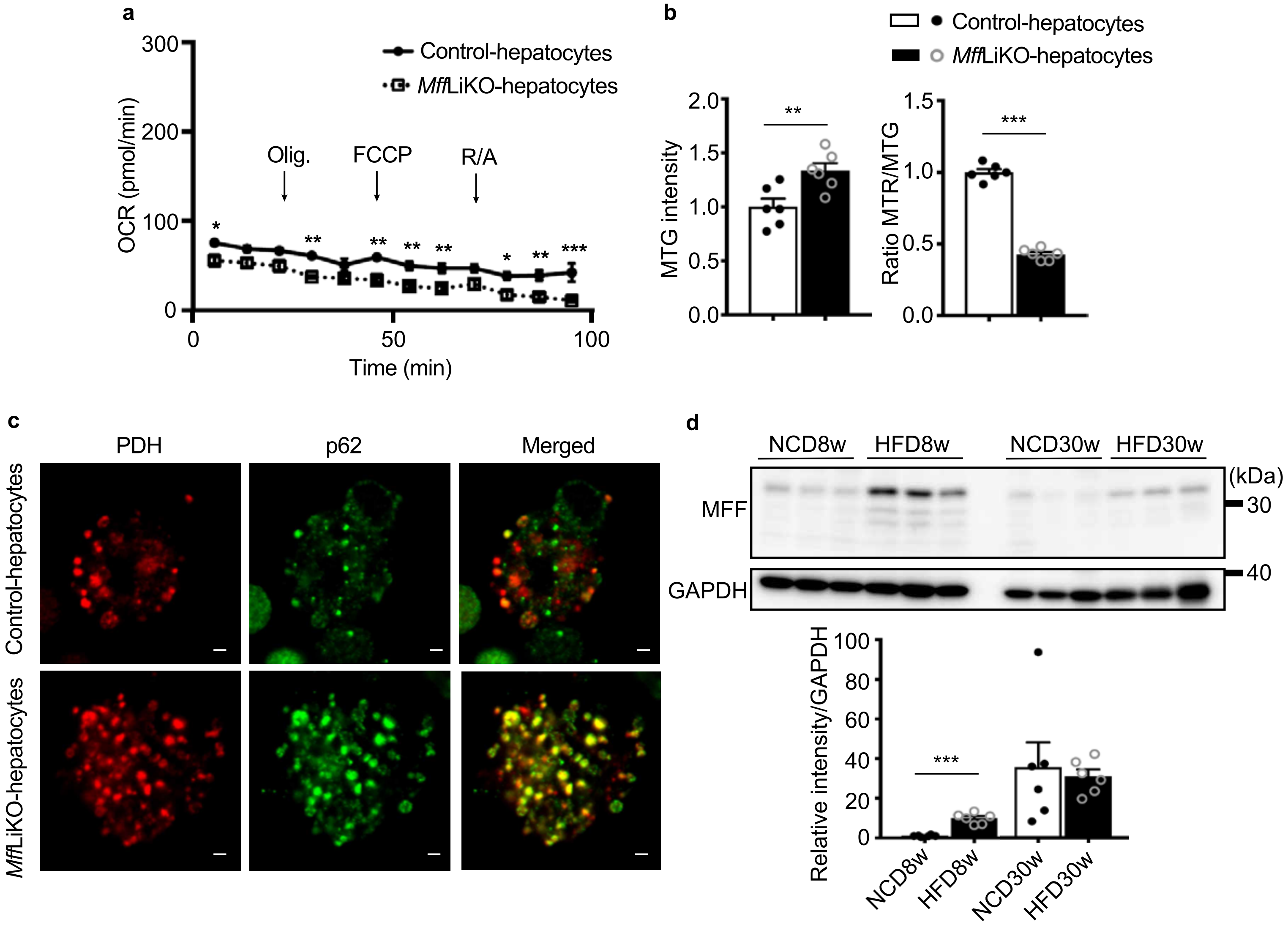

ESM Fig. 4 - Dysfunction of mitochondrial respiratory activity and mitophagy in *Mff*LiKO-hepatocytes from HFD-fed mice.

(**a**) Seahorse XF Cell Mito Stress Test data for OCR in *Mff*LiKO and control mouse hepatocytes from 30-week-old HFD-fed mice. Arrows indicate injections into media of the specific stressors oligomycin (Olig.), carbonyl cyanite-4 (trifluoromethoxy) phenylhydrazone (FCCP), and Rotenone/Antimycin A (R/A) (for each group n = 5).

(**b**) Mitochondrial membrane potential is shown by the fluorescence ratio of MTR to MTG in *Mff*LiKO and control mouse hepatocytes (for each group n = 6).

(**c**) Confocal immunofluorescence microscopy analysis of *Mff*LiKO and control mouse hepatocytes from 30-week-old HFD-fed mice using antibodies to pyruvate dehydrogenase (PDH) and p62. Scale bar = 1μm.

(**d**) Western blot analysis for protein expression of MFF in the livers from 8-and 30-week-old NCD- or HFD- fed control mice refed for 4 hours after 17-hour-fasting. Bar graphs show intensities of each protein band, quantified by densitometric analysis. Each intensity is normalised to GAPDH (for each group n = 3).

Values are expressed as means ± SEM. * p <0.05, ** p <0.01 and *** p <0.001, calculated by repeated measures Two-way ANOVA (**a**) or Student' s t test (**b, d**). Data are representative of two (**d**) or three (**a**, **b**) independent experiments .
